# Polyphenols ameliorate metabolic disorders by remodeling gut microbiota and regulating Nrf2/NF-κB signaling pathways

**DOI:** 10.3389/fmicb.2026.1862204

**Published:** 2026-07-29

**Authors:** Ying Zhou, Xinlin Zhou

**Affiliations:** College of Bioscience and Biotechnology, Hunan Agricultural University, Changsha, Hunan, China

**Keywords:** gut microbiota, metabolic diseases, Nrf2/NF-κB signaling pathways, oxidative stress, polyphenols

## Abstract

Intestinal barrier dysfunction, intestinal flora disorder and systemic chronic low-grade inflammation are key pathological processes that drive the progression of a variety of metabolic diseases (including obesity, type 2 diabetes and osteoporosis). Natural polyphenols have the ability to regulate intestinal microecology and regulate multi-target signal transduction, so they show significant metabolic regulation potential in dietary intervention strategies. In view of this, this review takes the pig model (which is highly similar to humans in terms of anatomy, physiology, immunology and intestinal microenvironment) as the research object to explore the intervention effect of polyphenols on metabolic diseases and its transformation clinical value. This review first explains the unique advantages and transformation application prospects of pigs as an ideal large animal model for human metabolic research. Subsequently, the system summarized the molecular mechanism of polyphenols to maintain intestinal homeosta by reshaping the core intestinal flora and promoting the production of short-chain fatty acids (SCFAs). In addition, this review also analyzes the regulatory effect of polyphenols in enhancing systemic antioxidant defense by activating the Nrf2/Keap1 pathway and inhibiting the release of pro-inflammatory factors by blocking the TLR4/NF-κB signaling pathway. By integrating the polyphenol intervention effect observed in the pig model and its potential molecular mechanism, this review provides reliable scientific evidence and theoretical reference for the development of dietary intervention strategies based on natural polyphenols and their subsequent clinical transformation.

## 1 Introduction

Metabolic diseases are one of the major public health threats to global human health. According to epidemiological estimates in 2025, more than 1.9 billion adults worldwide suffer from metabolic diseases, of which metabolic dysfunction-related fatty liver (MASLD) accounts for 29% of the population (Huang et al., 2025). The global shift to Western diet and sedentary lifestyle has led to a continuous increase in the incidence of obesity and metabolic disorders, greatly increasing the risk of complications such as cardiovascular disease and chronic kidney disease, thus exacerbating the burden on the global health care system. Metabolic diseases mainly include obesity, type 2 diabetes, osteoporosis and MASLD. They are characterized by high prevalence, early onset, and often accompanied by a variety of diseases (Chew et al., 2023). Their development is affected by the complex interaction of many factors such as genetic susceptibility, environmental stimulation, diet pattern and lifestyle factors. Its potential pathophysiological mechanism involves a variety of interrelated mechanisms, including intestinal flora dysregulation, chronic low-grade inflammation, oxidative stress, lipid metabolism disorders and abnormal signaling pathways (Cai et al., 2022; Kitada and Koya, 2021).

As an important digestive and immune organ, the intestine maintains barrier integrity and microbial homeostasis, which is crucial to regulating metabolic homeostasis. A complete intestinal barrier can prevent the invasion of pathogens and the absorption of harmful metabolites and endotoxins, maintain normal intestinal flora, maintain the physiological function of the mucus layer, and coordinate the epithelial barrier and the intestinal immune system, so as to avoid systemic chronic low-degree inflammation. Therefore, the integrity of the intestinal barrier is crucial to maintaining the whole body’s homeostasis (Zhang et al., 2023). By participating in key physiological processes, the intestinal flora directly affects glucose and lipid metabolism, energy balance and immune function, thus helping to maintain metabolic homeostasis (Ranson et al., 2025). Intestinal barrier dysfunction can cause endotoxin leakage, while intestinal flora disorder will disrupt the synthesis of microbial metabolites, including SCFAs. These changes work together to continuously activate inflammation and oxidative stress signaling pathways, leading to chronic low-degree inflammation, and ultimately promoting the progression of multiple metabolic disorders (Mishra et al., 2023). This pathological cascade reaction provides a key entry point for targeted intervention using natural bioactive compounds.

Natural bioactive compounds have become an important research direction in the field of physiological regulation. Among them, polyphenolic compounds are key natural drugs to intervene in metabolic diseases, and are also the core of metabolic disorder diet strategies at present. Polyphenols are widely found in nature, have good safety, and show a variety of biological activities, including antioxidant, anti-inflammatory and regulating intestinal flora. They can enhance the intestinal barrier function, maintain homeostasis, and reduce endotoxin displacement, thus reducing inflammatory reactions and oxidative stress, improving insulin resistance, and reducing fat accumulation (Sun et al., 2026; Fan et al., 2023). A variety of representative tea polyphenols play the role of intestinal protection and metabolic regulation through similar mechanisms, providing important experimental support for dietary intervention strategies. These findings highlight the potential importance and wide application prospects of tea polyphenols in combating metabolic diseases (Tang et al., 2022; Wu et al., 2021). To further clarify the *in vivo* effect and mechanism of action of polyphenols, it is necessary to use appropriate animal models for systematic evaluation. Such research is crucial for objectively evaluating its therapeutic potential and promoting clinical transformation. Therefore, this review uses the pig model to explore the mechanism of polyphenol intervention in metabolic diseases. We focus on how polyphenols reshape the intestinal flora and promote the production of SCFAs, and further clarify the molecular mechanism of activating the Nrf2 antioxidant defense system and inhibiting the NF-κB inflammatory signaling pathway. In a word, this review aims to provide a scientific basis for the clinical transformation and application of polyphenolic natural products.

## 2 The value of pig models in the study of metabolic diseases

### 2.1 Similarity with the human gastrointestinal tract

Among common experimental animals, pigs and humans are both real omnivores—this dietary characteristic gives them inherent similarities in nutritional needs and dietary adaptability, laying the foundation for pigs to become excellent experimental models. Research on the gastrointestinal microbiota and digestive physiology of experimental animals confirms that pigs are very close to humans in terms of intestinal nutrient conversion efficiency, meal transfer rhythm and overall digestive dynamics. This high degree of physiological consistency provides a solid foundation for the study of gastrointestinal physiology and nutritional metabolism (Mannino et al., 2022). Compared with the most commonly used experimental animal model, rodents, pigs have significant advantages in gastrointestinal microenvironment regulation. Their gastric acid secretion, intestinal pH environment, and the structure and function of the mucosal barrier are closer to humans. This high similarity allows pigs to accurately simulate the impact of dietary components on the gastrointestinal microenvironment and metabolic processes, making it an ideal model for the study of metabolic diseases associated with intestinal barrier dysfunction and nutritional metabolic disorders (Jia et al., 2024).

**Figure 1:**
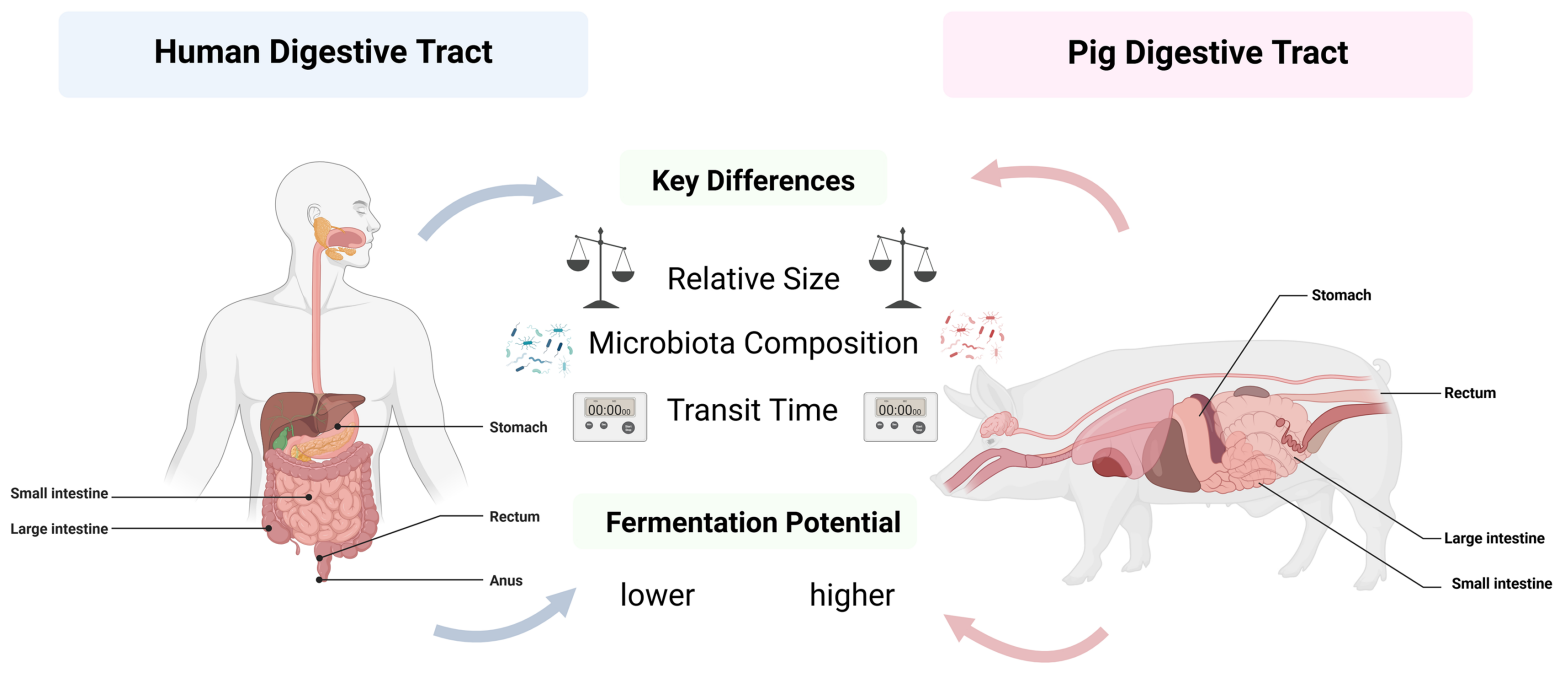
Comparison of the similarities and differences between human and pig intestines. The anatomical structure and physiological functions of the digestive tract of humans and pigs are highly conservative, so pigs are a mature model for studying human intestinal metabolism. The main differences between species are reflected in the relative size of the intestine, the composition of microbial communities, gastrointestinal transport time and fermentation capacity.

The value of pigs as a human biomedical research model is particularly significant in terms of their gastrointestinal anatomy and physiological and metabolic characteristics. Pigs and humans are highly homology in the morphology and tissue structure of gastrointestinal organs. Their digestive enzyme secretion pattern, intestinal nutrient transport efficiency and nutrient absorption mechanism are consistent with human physiological principles, and can stably reproduce metabolic disorders caused by special diets such as high-fat diet. In addition, the mature trajectory of the pig gastrointestinal system is highly similar to that of humans, which makes pigs an ideal model for longitudinal research, including long-term dietary intervention and functional evaluation of nutrients. The research results based on this model can highly simulate the real human physiological reaction, thus improving the reliability of the transformation of basic research results into clinical applications (Lunney et al., 2021). Recent comparative biomechanical research on gastrointestinal tissue further confirms that the biomechanical characteristics of the passive tissue of the pig stomach are highly consistent with the biomechanical characteristics of humans. This discovery provides direct evidence for pigs as tissue biomechanical models, further enhancing their importance in digestive physiology research (Holzer-Stock et al., 2025) (see Figure 1).

### 2.2 Similarity between the composition of intestinal flora and humans

The functional gene set and core metabolic pathways of pig intestinal flora are highly conservative with humans. Large-scale comparative macrogenomic research shows that there is a significant overlap between pigs and humans in the core microbial functional modules, including carbohydrate metabolism, amino acid metabolism and membrane transport. In terms of function, the similarity between pig intestinal flora and human intestinal flora is significantly higher than that of mice. These findings provide key data for pigs as an ideal large animal model for the study of intestinal microbiome (Xiao et al., 2016). The intestinal microbiota of pigs shows a clear spatial tissue structure in the intestinal segment, and its composition is closely related to the nutrient metabolism of the host. This feature can more realistically reflect the role of microbiota in energy absorption, nutrient conversion and metabolic regulation, thus providing a reliable animal model for the study of microbiome-related metabolic disorders (Song et al., 2022).

In terms of dietary intervention response, the metabolic phenotype changes of pigs and humans are highly similar. A high-fat diet will reshape the intestinal flora of pigs and induce obesity, insulin resistance and related metabolic abnormalities. Long-term high-fat feeding will further disrupt lipid metabolism. These diet-induced metabolic responses are highly similar to the physiological and metabolic characteristics of human obesity, which makes pigs a reliable model for simulating the occurrence and development of diet-related metabolic diseases (Rodríguez et al., 2020; Sanz-Fernandez et al., 2020). Due to this convergent metabolic response characteristics, the pig model effectively reproduces the influence of dietary factors on metabolic homeostasis and provides a suitable animal system for studying the potential mechanism of metabolic abnormalities caused by diet.

Therefore, the pig model is very suitable for systematically analyzing the mediating role of intestinal microbiota in the occurrence and development of metabolic diseases, and evaluating the regulatory role of polyphenolic compounds on intestinal microecosystems and their metabolic benefits (Anghel et al., 2024). The research results of this model are highly consistent with human physiological characteristics and have greater clinical transformation potential. Therefore, the pig model provides a reliable experimental platform for clarifying the mechanism of human metabolic diseases, developing nutritional interventions and designing prevention strategies. In addition, it also lays a solid theoretical foundation for future research in the field of precision nutrition and disease prevention.

### 2.3 Similarities in human immune structure and function

In addition to having advantages in anatomical structure, physiological function and intestinal microbiota, the immune system of pigs is highly conservative with the human immune system in terms of developmental characteristics, cell composition, organ structure and inflammatory signaling pathways. The composition of the intestinal mucosal immune system of pigs and its systemic immune response pattern are also highly similar to those of humans. This immunological similarity enables pigs to reproduce the occurrence and development of chronic low-degree inflammation associated with metabolic diseases (Li J. et al., 2024; Lagumdzic et al., 2022; Jeong et al., 2026). Compared with rodents, pigs are closer to humans in terms of immune cell subgroups, mucosal immune structure and systemic immune regulation network. It is worth noting that unlike rats and mice, pigs have a complete tonsils, which allows pigs to simulate human mucosal immunity and local immune responses more realistically (Pabst, 2020). These characteristics give the pig model a higher transformation value in immunorelated research. In addition, the regulatory mechanism of key inflammatory signaling pathways in pigs (including Toll-like receptors (TLR) and nuclear factor-κB (NF-κB) signaling pathways) can accurately reproduce the cadde of inflammatory reactions after the destruction of the intestinal barrier. This also makes the pig model suitable for evaluating the inhibitory effect of polyphenolic compounds on these inflammatory pathways (Luo et al., 2020; Chen J. et al., 2022).

The progress of gene editing technology has further expanded the use of pig models through the cultivation of immunodeficiency pigs. Wang’s group successfully constructed immunodeficient small pigs with RAG2 gene knockout. These small pigs showed T cell and B cell maturity defects and abnormal lymphatic organ development due to antigen receptor gene recombination damage (Wang et al., 2024). The model provides a large animal tool that can simulate human immunity more closely, so that immune cell function, mucosal immunity and inflammation-related mechanisms can be accurately studied. In addition, this model also helps to build tumor models and humanized animal models.

The pig model can also reliably reproduce chronic metabolic inflammation and endotoxemia caused by diet-induced obesity, and faithfully reproduce the pathological characteristics of human metabolic diseases (Starbæk et al., 2025). As a large animal model, pigs can carry out long-term dynamic immune monitoring and multi-tissue immune cell analysis. This provides a reliable experimental platform for analyzing the mechanism of immune dysfunction in metabolic diseases and evaluating the efficacy of anti-inflammatory interventions. Importantly, the pig model overcomes some key limitations of the rodent model, such as significant differences in immune structure and insufficient simulation of metabolic inflammation. Therefore, the research results of the pig model have higher conversion value. In addition, the model is also an ideal large animal platform to clarify the mechanism between metabolic disorders, intestinal barrier dysfunction and chronic inflammation (Table 1) (Sukhanov et al., 2023).

**Table 1:** Representative natural polyphenols and their mechanisms in alleviating metabolic disorders.

Polyphenol	Model	Induction method	Primary mechanism	Reference
Grape skin extract (e-Vitis)	Weaned piglets	Healthy model	Modulates bile acid metabolism; promotes production of beneficial microbial metabolites	Castillo et al. (2025)
Magnolol	Growing pigs	Healthy model	Reduces the abundance of *Desulfovibrio* in the colon	Li Y. et al. (2024)
Rice bran oil	Weaned piglets	LPS-induced injury	Increases jejunal villus height and villus height-to-crypt depth ratio; elevates CAT, SOD, and T-AOC levels; raises plasma levels of IgA, IgM, *β*-defensin-1, and lysozyme	Huang et al. (2023)
*Ampelopsis* *grossedentata*	Weaned piglets	Healthy model	Increases IL-10, IgG, and IgA expression; reduces IL-1β expression; increases GSH-Px, CAT, and SOD activity; increases the relative abundance of *Prevotella*	Liu et al. (2024)
Apple pomace or red-wine pomace diet	Weaned piglets	Healthy model	Stabilizes post-weaning villus morphology; reduces GALT activation, thereby decreasing inflammatory responses	Sehm et al. (2007)
Proanthocyanidins (PAC)	Growing pigs	Oral inoculation with fertilized *Ascaris suum* eggs	Increases the abundance of *Limosilactobacillus reuteri*; modulates genes associated with mucosal barrier function	Andersen-Civil et al. (2022)
Palm kernel cake oligosaccharides (PKCOS)	IPEC-J2 cell model	LPS-induced inflammatory injury	Activates the PI3K/MAPK signaling pathway; downregulates excessive expression of the pro-inflammatory factor TNF-α and Caspase-3; upregulates expression of SOD1, SOD2, and GPX4	Zeng et al. (2024)
Tannic acid (TA)	Weaned piglets	*E. coli* K88 infection	Inhibits the TLR4/MyD88/NF-κB signaling pathway; significantly increases serum concentrations of IgM and IgA; increases the abundance of *Ruminococcaceae*-associated bacteria	Zhou et al. (2026)
Sweet potato petiole and leaf diet	Aged minipig model	Natural aging	Increases the abundance of genera including *Muribaculaceae*, *Oscillibacter*, and *Desulfovibrio*	Sugai et al. (2025)
Gallnut tannic acid (GTA)	Early-pregnancy sow model	Healthy model	Increases T-SOD, CAT, and GSH-Px levels; reduces MDA content; significantly reduces the concentration of the pro-inflammatory cytokine IL-6	Wang et al. (2025)

These bioactive compounds show strong anti-inflammatory, antioxidant and insulin sensitization properties by targeting key signaling pathways. The emerging evidence highlights the structural diversity and multi-target regulatory role of polyphenols, providing a reliable framework for understanding how dietary plant chemicals link molecular interactions with systemic metabolic homeostasis in different experimental and clinical models.

## 3 Polyphenols remodel gut microbiota for metabolic homeostasis

Intestinal microbiota refers to various microbial communities, including bacteria, fungi and viruses, that have long been colonized in the intestines of humans and animals such as pigs. The number of this microbial community is about 10 times that of the host’s own cells. A healthy intestinal environment maintains the balance and diversity of this microbiota. On the other hand, the balanced microbiome supports the metabolic homeostasis of the host by enhancing intestinal immune function and tolerance, thus playing a key role in enhancing immunity, regulating intestinal endocrine function and affecting the production of host’s metabolic-related factors (Song et al., 2025; Luo et al., 2024).

### 3.1 Polyphenols improve metabolism by regulating intestinal flora

As a natural microecological regulator, polyphenolic compounds can reshape the composition of intestinal flora. Specifically, they selectively regulate the composition of microorganisms by inhibiting pathogenic bacteria and promoting the proliferation of beneficial bacteria and symbiotic bacteria (Vita et al., 2024).

#### 3.1.1 Inhibition of pathogenic bacteria

The significant inhibitory effect of polyphenols on intestinal Gram-negative pathogens has been directly verified in animal models. Chang’s team studied post-weaning diarrhea (PWD) induced by oral vaccination of pathogenic *E. coli* F18 using a 4-week-old weaned piglet as a model. An additional 0.1% of various plant additives were added to the intervention group, including bitter orange extract, grape peel extract and green tea. The results show that plant supplements significantly reduce the incidence of diarrhea, increase the ratio of villous height to crypt depth, and reduce the levels of tumor necrosis factor-α (TNF-α) and interleukin-6 (IL-6) in serum. These findings show that polyphenols effectively reverse the intestinal damage of pigs and maintain intestinal homeostasis by inhibiting the growth of pathogenic *E. coli*, thus preventing diarrhea after weaning (Chang et al., 2023). The antagonistic effect of polyphenols on the growth of pathogenic bacteria has been fully confirmed in cell-based *in vitro* studies. A study used the non-transformed pig intestinal epithelial cell line (IPEC-J2) isolated from the open intestine of newborn piglets that had never been weaned was used as an experimental model. The study uses lipopolysaccharides (LPS) to simulate gastrointestinal damage associated with weaning. IPEC-J2 cells were pretreated with 1 μg/mL or 2.5 μg/mL of blueberry crude phenol extract (BPE) for 24 h, and then stimulated with LPS for 6 h. The results show that BPE treatment not only significantly inhibits the growth of two intestinal pathogens—enterotoxin-producing *E. coli* (ETEC) and *Salmonella typhoid* (ST) at extremely low concentrations, but also protects the intestinal barrier function by increasing the abundance of mRNA of tightly connected proteins. These findings suggest that BPE indirectly improves the intestinal health of the host by inhibiting the proliferation of ETEC and ST, thus reducing their direct damage to the intestinal barrier (Nathan et al., 2024). Although the above animal and cellular studies provide evidence that polyphenols have antibacterial effects and protect the intestinal barrier, whether these findings can be reproduced in humans needs further translational studies. It is worth noting that some animal studies have adopted the model of weaned piglets. Given that pigs are very similar to humans in terms of intestinal structure, intestinal flora composition and immune structure, they are classic models for studying human intestinal infections and barrier dysfunction. Therefore, the selective regulation of polyphenols on intestinal microbiota observed in pig models provides valuable insights for understanding their application in human nutrition.

#### 3.1.2 Promotion of beneficial and commensal bacteria

In addition to inhibiting pathogenic bacteria, polyphenols can also selectively support beneficial bacteria and symbiotic bacteria in the intestine. A recent study modeled on intestinal barrier dysfunction induced by lipopolysaccharides (LPS) tested the effectiveness of plant polyphenolic acid (CA) as an intervention. Eighteen piglets were divided into three groups: control group (CON), LPS group and CA + LPS group (CAL). On the 21st and 28th days, the piglets in the LPS group and the CAL group were injected with O55:B5 *E. coli* LPS with a body weight of 80 μg/kg, respectively. The results show that CA not only increases the abundance of beneficial bacteria such as *Lactobacillus* and *Terrisporobacter*, but also reduces the abundance of *Rombus*, thus improving the diversity and composition of intestinal microorganisms. In addition, the study also found that CA reversed the reduction of 14 different bile acids and acetic acids induced by LPS. These findings show that CA can effectively improve intestinal morphology and barrier function, have a beneficial effect on intestinal flora and its metabolites, and promote the intestinal health of young pigs (Wen et al., 2024a). Similarly, Yu’s team used lactic acid bacteria derived from blackberries (*Lactobacillus plant* GBL 16 and 17) to ferment polyphenol-rich blackberry by-products. When this fermented product is fed to pigs, the abundance of beneficial bacteria in the intestine (including *Lactobacillus*, *Streptococcus*, *Weiss*, *Prevoella* and *Mycobacterium*) increases significantly. It is worth noting that the results showed that in the treatment group, the abundance of bacteria such as *Lactobacillus* and *Streptococcus* increased by 8.51 and 4.68%, respectively. These findings show that polyphenols can increase the proliferation of beneficial bacteria and symbiotic bacteria, thus positively regulating the intestinal flora and optimizing their composition, thereby regulating the host’s immunity and metabolic homeostasis (Yu et al., 2022). Based on the evidence of animal research, human fecal fermentation experiments *in vitro* further confirmed that polyphenols can selectively support beneficial bacteria and symbiotic bacteria in the intestine. In a study, fresh fecal samples were treated with mung bean seed skin extract (MSE) after 24 h of fermentation. MSE is a polyphenol-rich extract obtained by 50% ethanol pressurized liquid extraction (PLE). MSE significantly increased the abundance of *Bifidobacterium*, *Lactobacillus*, *Clostridium Pras* and *Prevoella*, while inhibiting the growth of potential pathogenic bacteria (such as *E. coli*). These findings demonstrate that MSE, which is rich in polyphenols, is expected to be a dietary supplement to maintain intestinal health and metabolic stability, and may help prevent or improve related metabolic diseases (Charoensiddhi et al., 2022). More importantly, clinical trials further confirm that polyphenols can selectively regulate the abundance of beneficial bacteria and symbiotic bacteria, thus improving the composition of intestinal flora. Del’s group conducted a dietary intervention study on 51 subjects using a high polyphenol diet (PR diet). The research portrait that the PR diet reduced the level of zonulin, an indirect marker of intestinal barrier function, and significantly increased the abundance of the family *Ruminococcaceae* and *Fecal bacillus*, thus improving the intestinal permeability of the subjects (Del Bo et al., 2021). Together, these studies emphasize the core role of polyphenols in improving intestinal function. These findings not only provide guidance for promoting the health of the elderly, but also point out the way for the wider application of polyphenol-rich diets in people of all ages in the future.

### 3.2 Polyphenols promote short-chain fatty acid production

The level of SCFAs is a key indicator of intestinal microecological homeosty. Among them, propionic acid and butyric acid are the key mediators for intestinal health, anti-inflammatory effects and metabolic improvement (Blaak et al., 2020). Polyphenols promote the production of short-chain fatty acids by reshaping the composition of intestinal flora, thus improving the structure and function of the intestinal barrier. SCFAs have become the focus of pig intestinal health research in recent years, promoting the rapid growth of relevant research. For example, in a study, polyphenol-rich grape seeds and grape skin extracts were added to the daily ration of weaned piglets in the first 2 weeks after weaning, and combined with a 0.1% low-dose functional amino acid mixture (including L-arginine, L-leucine and three other amino acids). The study found that this combination of functional amino acids and grape polyphenols can coordinate the intestinal flora and enhance the fermentation ability of intestinal microorganisms to dietary fiber, thus increasing the production of SCFAs such as butyric acid and propionic acid, and improving the absorption capacity of the intestine. In addition, the amino acid-polyphenol mixture significantly increases the abundance of pro-pro-*Lactobacillus* bacteria in the intestine and reduces the proportion of pathogenic bacteria (Beaumont et al., 2022). These research results clearly show that polyphenols are an effective strategy to maintain the intestinal homesta of weaned piglets by regulating intestinal flora and increasing the production of short-chain fatty acids. In addition, *in vitro* studies show that polyphenols play a crucial role in promoting the production of SCFAs and maintaining homeostasis. Loo’s team tested the combination of polyphenol-rich sugarcane extract (PRSE) and sugarcane fiber (SCFiber) using the *in vitro* pig manure fermentation model. They found that this combination not only significantly increased the bioavailable polyphenol content and antioxidant activity in the colon, but also promoted the generation of SCFAs, restoring the total SCFAs level to a level comparable to that of the control group. In addition, the PRSE-SCFiber combination also regulates the composition of fecal microorganisms, so that beneficial bacteria can occupy the ecological position and inhibit the colonation of pathogenic bacteria (Loo et al., 2023). Human manure fermentation experiments further support these findings. For example, polyphenol-rich propolis is famous for its antioxidant, anti-inflammatory and regulating intestinal flora. In the human manure fermentation model, poplar propolis polyphenols significantly raised the level of SCFAs produced by intestinal flora. This indicates that polyphenols increase the production of SCFAs by regulating intestinal microbial metabolism, thus improving the intestinal environment and intestinal health (Garzarella et al., 2022). These studies together show that polyphenols can effectively promote the enrichment and proliferation of bacteria that produce SCFAs in the intestines of humans and pigs, thus maintaining homeostasis, including by enhancing the intestinal barrier role.

**Figure 2:**
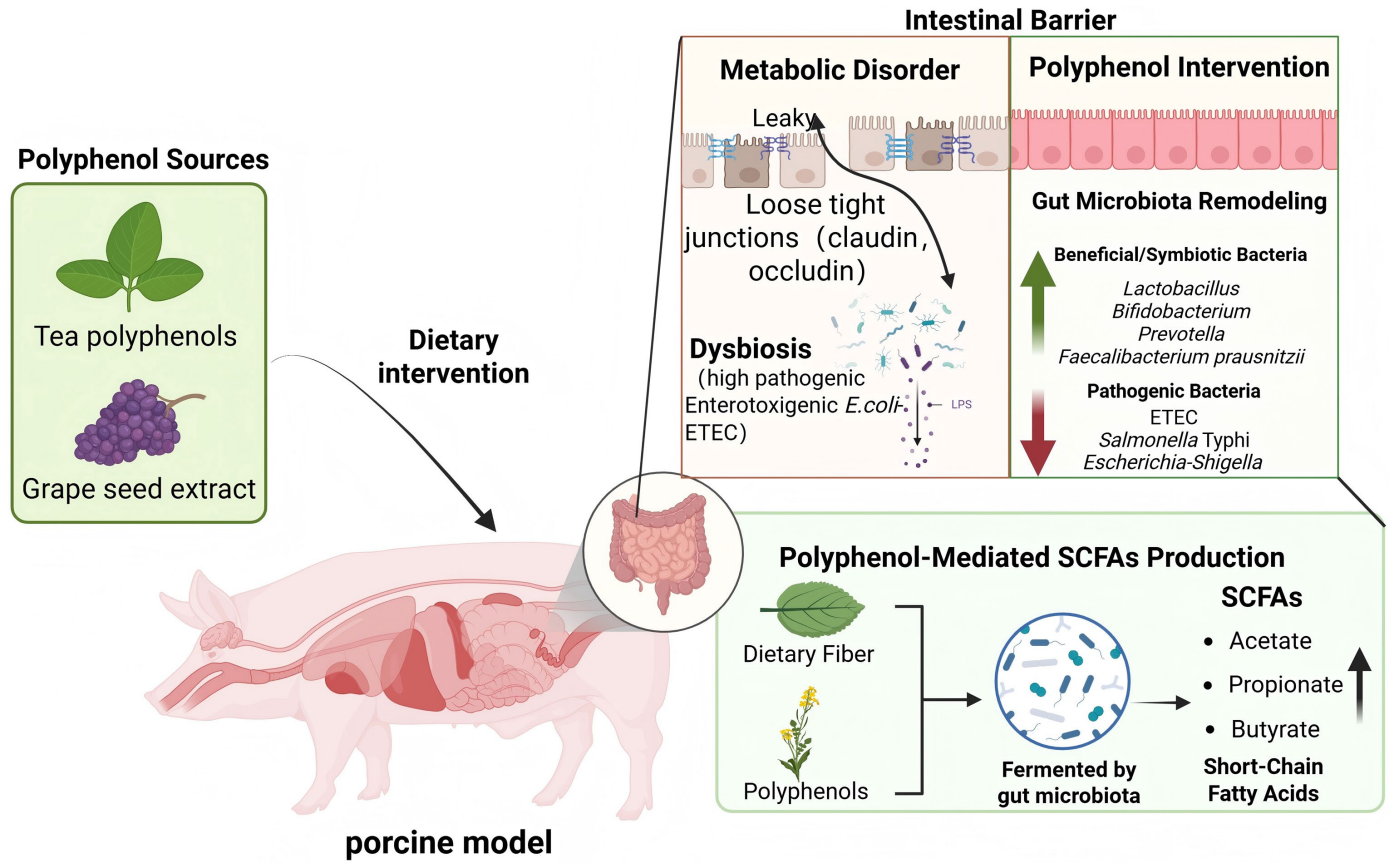
Polyphenols relieve metabolic disorders by regulating intestinal health. Polyphenols such as tea polyphenols and grape seed extracts can be used as effective dietary interventions to fight metabolic disorders in pig models. They repair the intestinal barrier role by enhancing the close connection and reverse the intestinal flora disorder. Polyphenols increase beneficial bacteria and inhibit pathogenic bacteria by reshaping the intestinal flora. They work together with dietary fiber to promote the production of SCFAs, ultimately enhancing systemic metabolic homeostasis and intestinal integrity.

Polyphenols can reshape the composition of intestinal flora and increase the production of SCFAs, and then regulate metabolism through a variety of downstream mechanisms. High-level SCFAs mainly activates the Nrf2 signaling pathway through two channels. First of all, butyric acid and other SCFAs, as histone deacetylase (HDAC) inhibitors, enhance histone acetylation in the Nrf2 promoter region, thus increasing the expression of Nrf2 mRNA at the transcription level. Secondly, SCFAs triggers the activation of the membrane receptor GPR41/43, initiates the intracellular kinase cascade reaction, and promotes the dissolation of Nrf2 and its inhibitory protein Keap1. The released Nrf2 is then transferred to the nucleus and combined with the antioxidant reaction element (ARE) to finally initiate the transcription of the downstream target gene HO-1. The activation of this pathway can significantly enhance the activity of antioxidant enzymes (including SOD, GSH-Px and CAT) while reducing the accumulation of MDA. It is worth noting that SCFAs-mediated Nrf2 activation can improve the antioxidant and anti-inflammatory ability of the host, and further regulate the antioxidant state of peripheral tissues, suggesting that there is a regulatory mechanism of microbial origin (Li et al., 2019). Although polyphenols can exert a strong anti-inflammatory effect by inhibiting the NF-κB signaling pathway, it is still not clear whether microbial metabolites directly target the pathway in the pig model, which represents the key direction of future research (see Figure 2).

## 4 Antioxidant and anti-inflammatory mechanisms of polyphenols

The core pathological feature of many metabolic diseases (including bone metabolic disorders, obesity and MASLD) is the synergy between oxidative stress (Abulikemu et al., 2023) and inflammatory reaction (Min et al., 2024). As a naturally occurring biologically active compound, polyphenols have considerable regulatory potential. A huge number of studies have described that polyphenols can grow cell oxidation defense by activating the Nrf2/Keap1 pathway, thus inducing the expression of endogenous antioxidant enzymes (Hseu et al., 2021). At the same time, polyphenols weaken the inflammatory response by blocking the TLR4/NF-κB pathway, thus halting the release of pro-inflammatory cytokines (Meng et al., 2023).

### 4.1 Activation of Nrf2/Keap1-mediated oxidative defense

The Nrf2/Keap1 pathway is a classic antioxidant stress defense mechanism and the core target of polyphenols. Under normal circumstances, Keap1 mediates the ubiquitinization and degradation of Nrf2. Polyphenols can induce Keap1 conformational changes, thus removing its inhibitory effect on Nrf2.

#### 4.1.1 Enhancement of endogenous antioxidant enzyme systems

After Nrf2 enters the nucleus, it binds to the antioxidant reaction element (ARE) to directly upregulate the gene expression that encodes endogenous antioxidant enzymes, thus giving full play to its oxidative defense function. Studies show that natural polyphenolic compounds such as fenalactyl ester (WDL) can significantly uptune the Keap1/Nrf2/ARE pathway, promote Nrf2 nuclear transposition, and thus initiate the transcription of downstream defense genes. Experimental data show that WDL treatment significantly improves the mRNA expression level of oxidative stress-related genes in pig embryos (Wang et al., 2022). In addition to WDL, other polyphenolic compounds also show a similar mechanism of regulating intestinal metabolic homeostasis. For example, a 28-day study using dichlorophenol (DQ) to induce intestinal oxidative damage, modeled on weaned piglets, showed that the intervention of polyphenolic compound hydroxytyrosol (HT) could protect the intestinal barrier from oxidative stress damage induced by DQ by enhancing antioxidant capacity. Given to the results of animal experiments, the author extends the research to cell experiments. Mechanism studies in IPEC-J2 cells show that hydroxytyrol activates the PI3K/Akt-Nrf2 signaling pathway and promotes mitochondrial autophagy, so that cells can more effectively neutralize superoxide anions produced under metabolic stress. This molecular mechanism maintains redox homeostasis and alleviates oxidative stress-related intestinal diseases (Wen et al., 2024b).

These studies together show that natural polyphenols reduce tissue damage caused by oxidative stress by regulating the Nrf2/Keap1 signal axis, enhancing mitochondrial activity and inducing mitochondrial autophagy (Court et al., 2024). In humans, pathological conditions are also associated with significant mitochondrial dysfunction and oxidative stress. Polyphenolic compounds provide a potential treatment direction for alleviating human metabolic diseases through dietary or drug supplementation by activating mitochondrial autophagy induced by Nrf2.

#### 4.1.2 Reducing free radicals and oxidative stress markers

The excessive accumulation of reactive oxygen (ROS) is a key factor leading to cell dysfunction during the development of metabolic diseases. In addition to enhancing the endogenous antioxidant enzyme system, polyphenols also have strong antioxidant properties, thanks to their unique chemical structure, which usually includes at least two benzene rings and one or more hydroxyl substituents, thus reducing oxidative stress (Grabska-Kobyłecka et al., 2023). The main active ingredient of catechin polyphenols is epigallocatechin-3-gallate (EGCG), which is a typical polyphenol derivative. Huang’s group treated pig oocytes with different concentrations of EGCG. They found that 10 μM EGCG treatment significantly reduced the level of ROS in mature oocytes, maintained the stability of the cell membrane, and significantly reduced the generation of malondialdehyde (MDA), thus reducing oxidative stress (Huang et al., 2021). Similarly, querthelin (Que) is a polyphenol with clear anti-inflammatory and antioxidant properties. Researchers tested it in a hydrogen peroxide (H2O2)-induced pig intestinal epithelial cell (IPEC-J2) damage model. The study found that qercetin can reduce the increase in the level of MDA in mild oxidized cells and restore the elevated MDA level, thus protecting IPEC-J2 cells from damage caused by oxidative stress and maintaining intestinal health (Chen et al., 2018).

In a word, polyphenolic substances can directly reduce the production of reactive oxygen (ROS), inhibit lipid peroxidation, and significantly reduce the level of oxidative damage marker malondialdehyde, thus activating an effective oxidative stress defense system (Guo et al., 2020). By reducing tissue damage caused by metabolic disorders, this mechanism provides a clear strategy for the prevention and treatment of metabolic diseases.

### 4.2 Inhibition of TLR4/NF-κB signaling

During the inflammatory reaction, Toll-like receptor 4 (TLR4) on the cell membrane—recognizes inflammatory stimuli, and then activates the downstream NF-κB signaling pathway, leading to the release of inflammatory cytokines (Li D. et al., 2022).

#### 4.2.1 Blocking LPS recognition complexes

TLR4/NF-κB signaling pathway is the key pathological mechanism that drives intestinal inflammation and damage. Polyphenols inhibit the binding of LPS and TLR4 receptor complexes, thus blocking the activation of NF-κB, effectively decreasing the release of inflammatory cytokines, and thus reducing inflammation. In a recent study, Zhang’s team used natural polyphenol chlorogenic acid (CGA) to intervene in the intestinal inflammation of weaned piglets. Experimental data shows that the addition of 200 mg/kg CGA to the daily ration can significantly reduce the concentration of pro-inflammatory cytokines in the ileum, thus reducing intestinal inflammation. Importantly, CGA reduces the expression level of TLR4 and its connecting protein MyD88, thus blocking the receptor’s recognition of LPS and inhibiting the phosphorylation and activation of downstream NF-κB, ultimately reducing intestinal inflammation in piglets (Zhang et al., 2024). A research demonstrated that the combination of natural polyphenols curcumin and resveratrol (HRC) can reduce intestinal inflammation and improve intestinal immune function. The study randomly divided 180 weaned piglets (21 ± 2 days old) into multiple groups. The piglets in the HRC group were fed a daily ration with 300 mg/kg of resveratrol and curcumin for 28 days. The results showed that HRC treatment reduced the expression of TLR4 protein in the ileum, inhibited the release of pro-inflammatory cytokines such as interleukin-1β (IL-1β) and tumor necrosis factor-*α* (TNF-α), increased the secretion of immunoglobulin, and finally restored intestinal immune function (Gan et al., 2019). In humans and weaned piglets, inhibiting the activation of the TLR4/NF-κB pathway induced by LPS can effectively reduce inflammation, maintain systemic homeostasis, and prevent the occurrence of metabolic diseases.

#### 4.2.2 Blocking NF-κB p65 phosphorylation and nuclear entry

After inflammatory stimulation, p65 undergoes phosphorylation, promoting the rapid activation and nuclear transposition of NF-κB, leading to the production of a massive number of pro-inflammatory cytokines and strong inflammatory reactions (Du et al., 2023). Polyphenols are natural organic compounds widely found in plants. Chen and colleagues verified through experiments that polyphenols alleviate inflammatory injury by inhibiting the IKK/IκB signaling axis, markedly reducing NF-κB p65 phosphorylation and blocking its nuclear translocation (Chen S. et al., 2022). Li’s team divided 72 weaned piglets into groups and pretreated them with the polyphenolic compound pterostilbene (PT), a 3,5-dimethyl ether derivative, for 14 days prior to LPS-induced liver injury and immune dysfunction. Following the feeding period, piglets received intraperitoneal LPS injections. The results showed that PT not only reduced the expression levels of IL-1β and IL-6 but also inhibited the nuclear translocation of NF-κB p65, NLRP3, and cleaved caspase-1, thereby reversing LPS-induced apoptosis and inflammation (Li Y. et al., 2022). NF-κB signal conduction and inhibition of p65 phosphorylation are the key mechanisms for polyphenols to alleviate inflammatory reactions.

**Figure 3:**
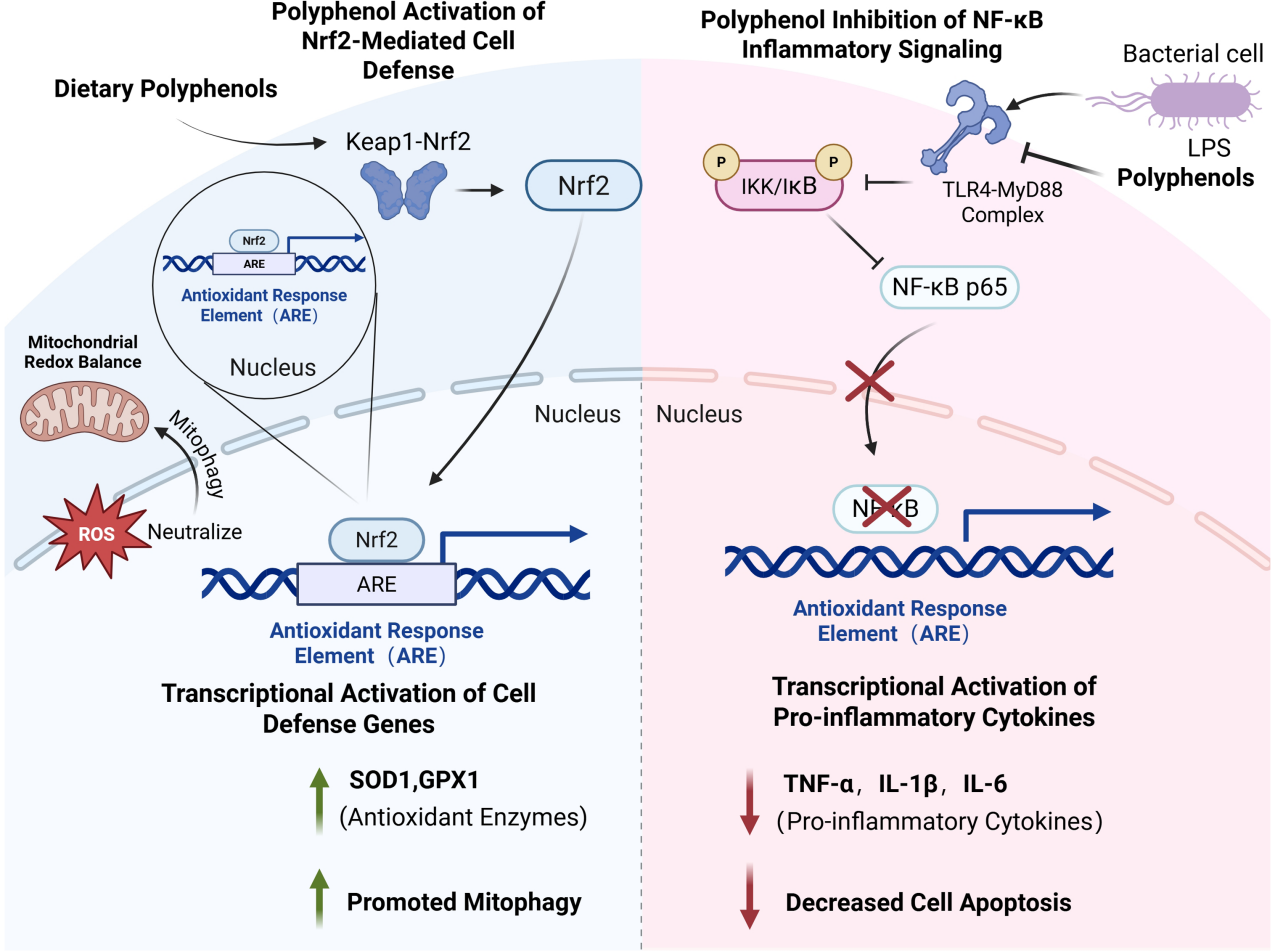
The mechanism of polyphenols to relieve oxidative stress and inflammation. Dietary polyphenols play a dual protective role by activating the Nrf2-Keap1 pathway and inhibiting the NF-κB signaling pathway. They promote Nrf2 nuclear transposition, enhance the expression of antioxidant enzymes, and induce mitochondrial autophagy, thus neutralizing ROS to maintain mitochondrial redox balance. At the same time, polyphenols block the activation of TLR4 induced by LPS, inhibit the phosphorylation of IKK/IκB, and inhibit the expression of downstream pro-inflammatory cytokines. This synergistic regulation effectively reduces cell oxidative damage and prevents cell apoptosis.

It is worth noting that inhibiting phosphorylation and nuclear transposition of NF-κB p65 is not an isolated anti-inflammatory mechanism, usually accompanied by an improvement in redox balance. By blocking the phosphorylation and nuclear transposition of the key NF-κB sub-unit p65, polyphenols significantly inhibit the production of excessive ROS induced by inflammatory cytokines, thus reducing tissue damage (Zhang et al., 2025). Similarly, this mechanism can effectively reduce chronic inflammation of the human intestine and liver, improve metabolic health, and maintain whole body homeostasis (see Figure 3).

## 5 Conclusion

### 5.1 Limitations and future perspectives

Although the pig model has shown significant value in the study of metabolic diseases, its practical application is still constrained by some inherent limitations. First of all, the cost of purchasing and raising pigs is hundreds of times higher than that of rodent models, and such a huge economic burden limits large-scale experiments and biological repeat verification. Secondly, sterile pigs often used in specific microbiology research need to strictly maintain sterile breeding conditions throughout the experimental process to eliminate bacterial and fungal contamination and physiological abnormalities or adverse reactions. This strict breeding system consumes a lot of manpower and material resources, which greatly limits the accessibility and applicability of the sterile pig model compared with the traditional experimental model (Hara et al., 2018). Although the genetic background of experimental pigs is relatively homogeneous, there are still significant phenotypic individual differences between different experimental subjects, which brings significant limitations to the study of intestinal flora and disease progression. In addition, as large mammals, pigs need to undergo stricter ethical scrutiny and are subject to stricter animal welfare regulations than rodents. In terms of life expectancy, pigs live much shorter than humans, which limits their ability to simulate human age-related diseases. Finally, the composition of the intestinal flora of experimental pigs is very susceptible to the influence of feed formula and feeding environment. So far, the research community has not established a common baseline reference standard, which constitutes a major obstacle to experimental repeatability (Pirolo et al., 2021).

Given that pigs are highly similar to humans in terms of organ size, tissue structure, metabolic function and immunity, the pig model is one of the most important animal models for the study of human metabolic diseases. Compared with classic rodent models such as mice, pigs show higher similarities with humans in key pathophysiological processes, including bone remodeling rate, fat deposition patterns and lipid metabolism, thus they are closer to human clinical phenotype (Zheng et al., 2024). This advantage effectively overcomes the limitations of the rodent model, which cannot fully reproduce the complex pathophysiological process of human diseases. It is worth noting that with the continuous progress of gene editing technology, the introduction of exogenous genes in pigs or precise genome modification can build a genetically modified pig model that can simulate the characteristics of human diseases better than traditional model methods, thus laying the experimental foundation for drug research and development.

In recent years, natural polyphenol products have become a research hotspot for human metabolic diseases because of their anti-inflammatory, antioxidant and anti-cancer properties (Alldritt et al., 2019). However, the poor water solubility of polyphenol compounds seriously limits their intestinal absorption efficiency. Only a small number of complete polyphenolic compounds can be absorbed by the epithelial cells of the small intestine and enter the systemic circulation, while most of them rely on intestinal microbial metabolism to play pharmacological effects. In addition, polyphenol compounds have poor stability in the body, and extensive metabolic transformation will occur in the process of absorption and tissue distribution, thus changing their target specificity and biological activity. Moreover, there are significant differences in the metabolic capacity of polyphenols between individual pigs, which leads to differences in treatment and antioxidant effects. It is worth noting that the reactive oxygen removal and antioxidant properties of polyphenolic compounds are dose-dependent: low-dose treatment has little effect, while excessive treatment may trigger oxidation-promoting reactions. Coupled with its low bioavailability, these limiting factors greatly increase the difficulty of determining the optimal dose and hinder the conversion and application of polyphenolic compounds in clinical trials (Posadino et al., 2019).

### 5.2 Concluding remarks

This review summarizes the latest research progress of natural polyphenolic compounds to improve metabolic homeostasis through multi-target mechanisms, including intestinal flora remodeling, activation of antioxidant pathways and inhibition of inflammatory signal transduction. We focus on the mechanism of polyphenol compounds regulating the abundance of core intestinal flora, enhancing the endogenous antioxidant enzyme system and blocking the TLR4/NF-κB pathway in the pig model. Together, these findings support the beneficial role of polyphenols in maintaining intestinal barrier function and metabolic balance.

In a word, because its physiological structure is highly similar to that of humans, the pig model has a significant advantage in evaluating the efficacy and molecular mechanism of natural polyphenol products. In-depth research using the pig model can not only simulate the mechanism of action of polyphenols in the body more realistically, but also provide more scientific and reliable experimental evidence for the application of polyphenol-based functional foods or natural drugs in the treatment of human metabolic diseases.
